# Crystal structure and DFT study of (*E*)-2,6-di-*tert*-butyl-4-{[2-(pyridin-2-yl)hydrazin-1-yl­idene)meth­yl}phenol

**DOI:** 10.1107/S2056989017011707

**Published:** 2017-09-12

**Authors:** Md. Serajul Haque Faizi, Ashanul Haque, Mustafa Dege, Necmi Dege, Maria L. Malysheva

**Affiliations:** a Department of Chemistry, College of Science, Sultan Qaboos University, PO Box 36 Al-Khod 123, Muscat, Sultanate of , Oman; b Spraying Systems Company Turkey, Esentepe Mah. Kore Şehitleri Cad. Kaya Aldoğan Sok., Serhan apt. No:3 Daire:3 Şişli / İstanbul, Turkey; c Ondokuz Mayıs University, Arts and Sciences Faculty, Department of Physics, Atakum 55139 Samsun, Turkey; d Department of Chemistry, Taras Shevchenko National University of Kyiv, 64, Vladimirska Str., Kiev 01601, Ukraine

**Keywords:** crystal structure, hydrazine, 2- hydrazino­pyridine, 3,5-di-*tert*-butyl-4-hy­droxy­benzaldehyde, hydrogen bonding, Schiff base

## Abstract

The title compound, C_20_H_27_N_3_O, was synthesized by condensation reaction of 3,5-di-*tert*-butyl-4-hy­droxy­benzaldehyde and 2-hydrazinyl­pyridine, and crystallizes in the centrosymmetric monoclinic space group *C*2/*c*. The conformation about the C=N bond is *E*. The dihedral angle between the rings is 18.1 (3)°. An inter­molecular N—H⋯N hydrogen bond generates an *R*_2_^2^(8) ring motif. In the crystal, N—H⋯N hydrogen bonds connect pairs of mol­ecules, forming dimers. Density functional theory (DFT) optimized structures at the B3LYP/6–311 G(d,p) level are compared with the experimentally determined mol­ecular structure in the solid state.

## Abstract 2

The title Schiff base was synthesized *via* the condensation reaction of 3,5-di-*tert*-butyl-4-hy­droxy­benzaldehyde and 2-hydrazinyl­pyridine and crystallized with a single mol­ecule in the asymmetric unit. The conformation about the C=N bond is *E*. In the crystal, N—H⋯N hydrogen bonds connect pairs of mol­ecules into dimers. In addition, weak C—H⋯O hydrogen bonds and C—H⋯π inter­actions are observed.

## Chemical context   

Sterically hindered phenol anti-oxidants are widely used in polymers and lubricants. They can protect polymers by increasing both their process stability and their long-term stability against oxidative degradation (Yamazaki & Seguchi, 1997 ▸; Silin *et al.*, 1999 ▸). Hydrazones and Schiff bases have attracted much attention for their excellent biological properties, especially for their potential pharmacological and anti­tumor properties (Küçükgüzel *et al.*, 2006 ▸; Khattab, 2005 ▸; Karthikeyan *et al.*, 2006 ▸; Okabe *et al.*, 1993 ▸). Furthermore, 3,5-di-*tert*-butyl-2-hy­droxy­benzaldehyde-derived Schiff bases shows proton tautomerization, which plays an important role in many fields of chemistry and biochemistry. The tautomerization in salicylideneanilines has been the subject of particular inter­est because it is closely related to thermochromism and photochromism. While salicylideneanilines are widely used as precursor compounds for the design of various type new metal complexes, they are also convenient model compounds for studying theoretical aspects of coordination chemistry and photochemistry, as well as for designing mol­ecular architectures by means of mol­ecular motifs capable of hydrogen-bond formation. The present work is a part of an ongoing structural study of Schiff bases and their utilization in the synthesis of quinoxaline derivatives (Faizi *et al.*, 2016*a* ▸), fluorescence sensors (Faizi *et al.*, 2016*b* ▸) and azo­imine compounds (Faizi *et al.*, 2015 ▸, 2017 ▸). We report herein on the synthesis and crystal structure and DFT computational calculation of the new title Schiff base compound with a sterically hindered phenol, (I). The results of calculations by density functional theory (DFT) on (I) carried out at the B3LYP/6-311 G(d,p) level are compared with the experimentally determined mol­ecular structure in the solid state.
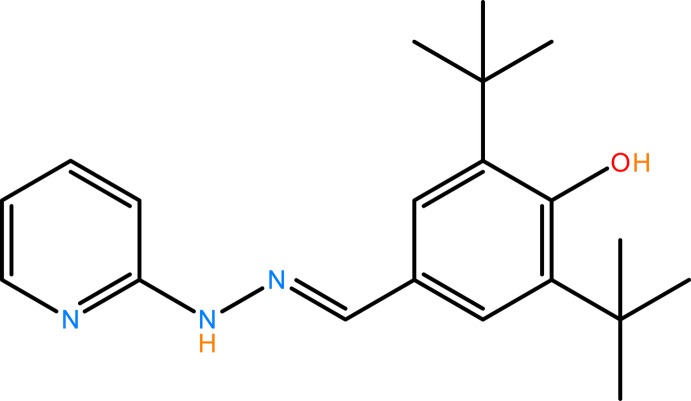


## Structural commentary   

**Figure 1:**
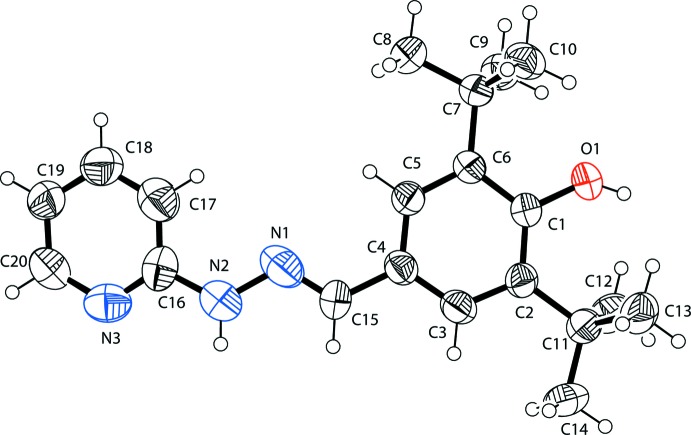
The mol­ecular structure of the title compound, with the atom labelling. Displacement ellipsoids are drawn at the 40% probability level.

The mol­ecular structure of (I), shown in Fig. 1 ▸, is not planar, with the dihedral angle between the pyridyl and *tert*-butyl substituted benzene rings being 18.19 (3)°. The N—N and N—C bond lengths are of 1.396 (7) and 1.253 (7) Å, respectively, indicate single- and double-bond character for these bonds. The C1—O1 bond length of 1.370 (6) Å indicates single-bond character. The conformation about the C15=N1 bond is *E* with an N2—N1—C15—C4 torsion angle of 177.9 (5)°. Bond distances for (I) are comparable to those found in closely related structures (Fun *et al.*, 2013 ▸). It appears that the hy­droxy group is prevented from forming a hydrogen bond because of steric hindrance by the *tert*-butyl groups.

## Supra­molecular features   

**Figure 2:**
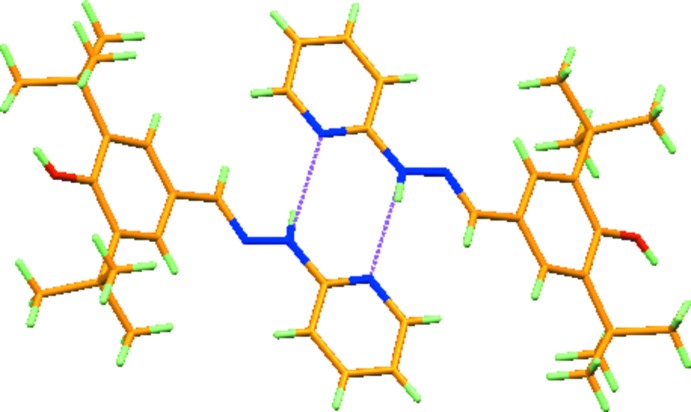
Mol­ecules of the title compound forming a dimer through N—H⋯N hydrogen bonds, generating an 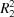
(8) ring motif.

**Figure 3:**
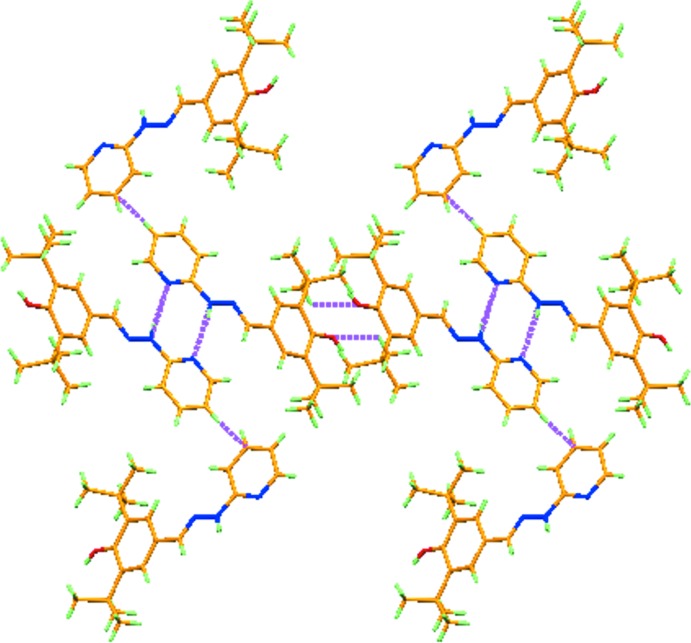
Part of the structure exhibiting weak C—H⋯O hydrogen bonds and C—H⋯π inter­actions (shown as dashed lines) along *a* axis.

In the crystal, mol­ecules are connected by pairs of N—H⋯N hydrogen bonds (Fig. 2 ▸, Table 1 ▸), forming dimers with graph set 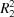 (8). In addition, weak C—H⋯O hydrogen bonds and C—H⋯π interactions connect the dimers, forming chains along [100] (Fig. 3 ▸). There are no other significant inter­molecular contacts present.

## DFT study   

The DFT quantum-chemical calculations were performed at the B3LYP/6-311 G(d,p) level (Becke, 1993 ▸) as implemented in *GAUSSIAN09* (Frisch *et al.*, 2009 ▸). DFT structure optimization of (I) was performed starting from the X-ray geometry and the values compared with experimental values (see Table 2 ▸). From these results we can conclude that basis set 6-311 G(d,p) is well suited in its approach to the experimental data.

**Figure 4:**
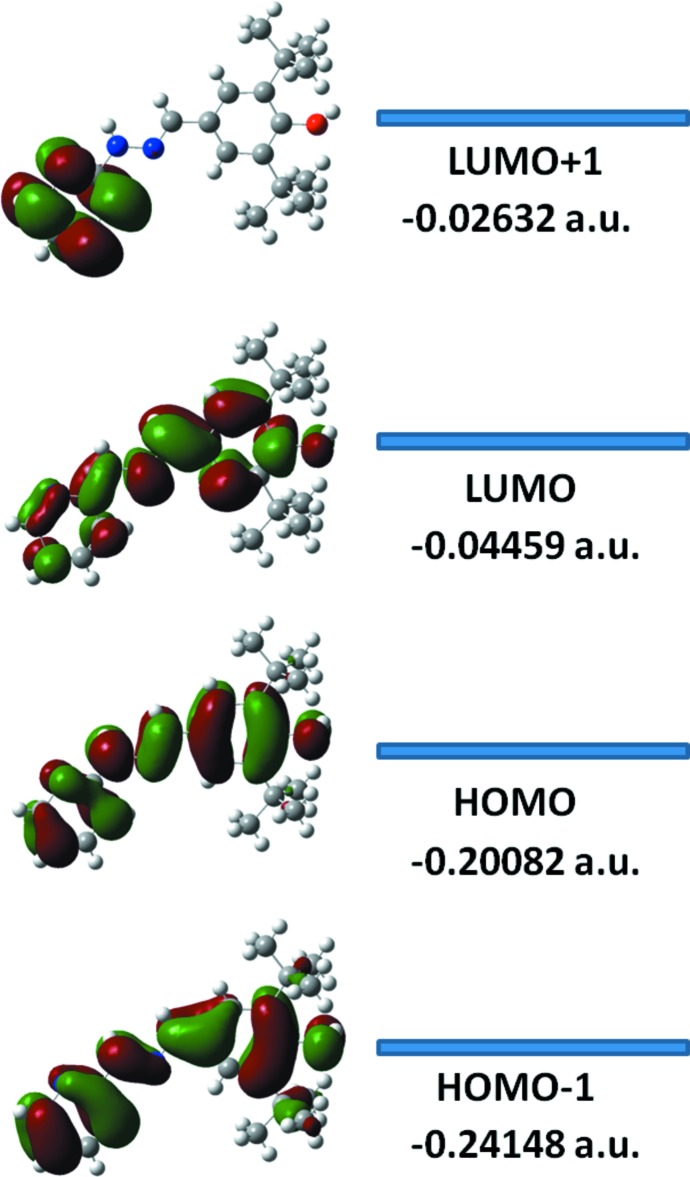
Electron distribution of the HOMO-1, HOMO, LUMO and LUMO+1 energy levels for the title compound.

The DFT study of (I) shows that the HOMO and LUMO are localized in the plane extending from the whole pyridine ring to the phenol ring. The electron distribution of the HOMO-1, HOMO, LUMO and the LUMO+1 energy levels are shown in Fig. 4 ▸. The HOMO mol­ecular orbital exhibits both σ and π character, whereas HOMO-1 is dominated by π-orbital density. The LUMO is mainly composed of σ-density while LUMO+1 has both σ and π electronic density. The HOMO–LUMO gap was found to be 0.1562 a.u. and the frontier mol­ecular orbital energies, *E*_HOMO_ and *E*_LUMO_ are −0.201 and −0.045 a.u., respectively.

## Database survey   

There are very few examples of similar compounds in the literature. To the best of our knowledge, the similar compound synthesized by (Cuadro *et al.*, 1998 ▸) for biological evaluation of 5-lipoxygenase inhibitors has not been structurally characterized. Two very similar compounds have been reported, one synthesized from 2-hydrazinyl­pyridine and 4-*tert*-butyl-2,6-di­formyl­phenol (Li *et al.*, 2013 ▸) as a fluorescent chemosensor for Zn^II^ and applications in live cell imaging. The other compound is the Schiff base 2,4-di-*tert*-butyl-6-{[2-(pyridin-2-yl)hydrazono]meth­yl}phenol used for stabilization of oxidovanadium(IV) (Kundu *et al.*, 2013 ▸).A search of the Cambridge Structural Database (CSD, Version 5.37, update May 2016; Groom *et al.*, 2016 ▸) shows that these compounds have not been characterized by X-ray diffraction.

## Synthesis and crystallization   

A mixture of 3,5-di-*tert*-butyl-4-hy­droxy­benzaldehyde 0.100 g (0.427 mmol) and 2-hydrazinyl­pyridine 0.046 g (0.427 mmol) in methanol was refluxed for 3 h in the presence of a catalytic amount of glacial acetic acid. After cooling, the red-coloured precipitate was washed with hot methanol several times, and then dried, giving a red-coloured shiny crystalline compound in 86% yield (0.120 g). Red block-like crystals of the title compound were obtained by slow evaporation of a solution in di­chloro­methane and ethanol (5:1 *v*/*v*).

## Refinement   

Crystal data, data collection and structure refinement details are summarized in Table 3 ▸. All C-bound hydrogen atoms were included in calculated positions with C—H = 0.93 (aromatic) or 0.96 Å (methyl­ene) and allowed to ride, with *U*_iso_(H) = 1.2*U*_eq_(C). The N-bound H atom was located in a difference-Fourier map but was also allowed to ride in the refinement with N—H = 0.86 Å and *U*_iso_(H) = 1.2*U*_eq_(N).

## Supplementary Material

Crystal structure: contains datablock(s) I. DOI: 10.1107/S2056989017011707/hg5492sup1.cif

Structure factors: contains datablock(s) I. DOI: 10.1107/S2056989017011707/hg5492Isup2.hkl

CCDC reference: 1567740

Additional supporting information:  crystallographic information; 3D view; checkCIF report

## Figures and Tables

**Table 1:** Hydrogen-bond geometry (Å, °)

*D*—H⋯*A*	*D*—H	H⋯*A*	*D*⋯*A*	*D*—H⋯*A*
N2—H2⋯N3^i^	0.86	2.23	3.062 (8)	162

Symmetry code: (i) 
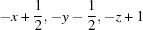
.

**Table 2:** Comparison of selected observed (X-ray data) and calculated (DFT) geometric parameters (Å, °)

Parameter	X-ray	B3LYP/6–311G(d,p)
O1—C1	1.370 (6)	1.370
C15—N1	1.253 (7)	1.252
N3—C20	1.386 (8)	1.386
N1—N2	1.396 (7)	1.395
N3—C16	1.292 (8)	1.292
C16—N2—N1	122.6 (6)	122.7
C15—N1—N2	118.8 (6)	118.9
N1—C15—C4	121.9 (6)	121.9
N2—N1—C15—C4	177.9 (5)	177.8

**Table 3:** Experimental details

Crystal data	
Chemical formula	C_20_H_27_N_3_O
*M*_r_	325.44
Crystal system, space group	Monoclinic, *C*2/*c*
Temperature (K)	296
*a*, *b*, *c* (Å)	29.5091 (15), 6.2270 (4), 20.2703 (10)
β (°)	91.130 (4)
*V* (Å^3^)	3724.0 (4)
*Z*	8
Radiation type	Mo *K*α
μ (mm^−1^)	0.07
Crystal size (mm)	0.33 × 0.24 × 0.08

Data collection	
Diffractometer	Stoe IPDS 2
Absorption correction	Integration (*X-RED32*; Stoe & Cie, 2002 ▸)
*T*_min_, *T*_max_	0.978, 0.994
No. of measured, independent and observed [*I* > 2σ(*I*)] reflections	17357, 3468, 1430
*R*_int_	0.097
(sin θ/λ)_max_ (Å^−1^)	0.606

Refinement	
*R*[*F* ^2^ > 2σ(*F* ^2^)], *wR*(*F* ^2^), *S*	0.101, 0.321, 0.96
No. of reflections	3468
No. of parameters	222
No. of restraints	4
H-atom treatment	H atoms treated by a mixture of independent and constrained refinement
Δρ_max_, Δρ_min_ (e Å^−3^)	0.95, −0.34

Computer programs: *X-AREA* and *X-RED32* (Stoe & Cie, 2002 ▸), *SHELXT* (Sheldrick 2015*a* ▸), *SHELXL2016* (Sheldrick, 2015*b* ▸), *ORTEP-3 for Windows* and *WinGX* (Farrugia, 2012 ▸) and *PLATON* (Spek, 2009 ▸).

## supplementary crystallographic information

### Crystal data

**Table:**

C_20_H_27_N_3_O	*F*(000) = 1408
*M**_r_* = 325.44	*D*_x_ = 1.161 Mg m^−^^3^
Monoclinic, *C*2/*c*	Mo *K*α radiation, λ = 0.71073 Å
*a* = 29.5091 (15) Å	Cell parameters from 10906 reflections
*b* = 6.2270 (4) Å	θ = 1.4–26.8°
*c* = 20.2703 (10) Å	µ = 0.07 mm^−^^1^
β = 91.130 (4)°	*T* = 296 K
*V* = 3724.0 (4) Å^3^	Stick, red
*Z* = 8	0.33 × 0.24 × 0.08 mm

### Data collection

**Table:**

Stoe IPDS 2 diffractometer	3468 independent reflections
Radiation source: sealed X-ray tube, 12 x 0.4 mm long-fine focus	1430 reflections with *I* > 2σ(*I*)
Plane graphite monochromator	*R*_int_ = 0.097
Detector resolution: 6.67 pixels mm^-1^	θ_max_ = 25.5°, θ_min_ = 1.4°
rotation method scans	*h* = −35→35
Absorption correction: integration (X-RED32; Stoe & Cie, 2002)	*k* = −7→7
*T*_min_ = 0.978, *T*_max_ = 0.994	*l* = −24→24
17357 measured reflections	

### Refinement

**Table:**

Refinement on *F*^2^	4 restraints
Least-squares matrix: full	Hydrogen site location: mixed
*R*[*F*^2^ > 2σ(*F*^2^)] = 0.101	H atoms treated by a mixture of independent and constrained refinement
*wR*(*F*^2^) = 0.321	*w* = 1/[σ^2^(*F*_o_^2^) + (0.1794*P*)^2^] where *P* = (*F*_o_^2^ + 2*F*_c_^2^)/3
*S* = 0.96	(Δ/σ)_max_ < 0.001
3468 reflections	Δρ_max_ = 0.95 e Å^−^^3^
222 parameters	Δρ_min_ = −0.34 e Å^−^^3^

### Special details

**Table:**

Geometry. All esds (except the esd in the dihedral angle between two l.s. planes) are estimated using the full covariance matrix. The cell esds are taken into account individually in the estimation of esds in distances, angles and torsion angles; correlations between esds in cell parameters are only used when they are defined by crystal symmetry. An approximate (isotropic) treatment of cell esds is used for estimating esds involving l.s. planes.

### Fractional atomic coordinates and isotropic or equivalent isotropic displacement parameters (Å^2^)

**Table:**

	*x*	*y*	*z*	*U*_iso_*/*U*_eq_	
O1	0.44818 (13)	0.9043 (6)	0.53805 (18)	0.0870 (13)	
C6	0.40894 (16)	0.6560 (8)	0.4671 (2)	0.0602 (12)	
C1	0.41473 (17)	0.7532 (8)	0.5303 (2)	0.0643 (13)	
N3	0.25835 (18)	−0.3445 (11)	0.4118 (3)	0.0956 (15)	
C5	0.37623 (17)	0.4983 (8)	0.4620 (2)	0.0653 (13)	
H5	0.371669	0.430688	0.421532	0.078*	
C2	0.38832 (17)	0.6977 (7)	0.5845 (2)	0.0616 (13)	
C7	0.43754 (17)	0.7232 (8)	0.4082 (2)	0.0652 (13)	
C4	0.34989 (16)	0.4362 (8)	0.5145 (2)	0.0630 (13)	
C3	0.35632 (17)	0.5396 (8)	0.5744 (2)	0.0682 (13)	
H3	0.338180	0.500452	0.609405	0.082*	
C11	0.39633 (18)	0.8015 (8)	0.6539 (2)	0.0692 (14)	
C16	0.2803 (2)	−0.1665 (11)	0.4050 (3)	0.0854 (18)	
C15	0.31797 (19)	0.2599 (9)	0.5093 (3)	0.0753 (15)	
H15	0.299604	0.229578	0.544903	0.090*	
N1	0.31443 (18)	0.1472 (10)	0.4583 (3)	0.1040 (17)	
C9	0.48786 (17)	0.6685 (9)	0.4218 (3)	0.0788 (15)	
H9A	0.498240	0.741762	0.460985	0.118*	
H9B	0.491083	0.516377	0.427934	0.118*	
H9C	0.505562	0.713463	0.385090	0.118*	
C14	0.3634 (2)	0.7150 (10)	0.7038 (3)	0.0892 (18)	
H14A	0.367140	0.562344	0.707671	0.134*	
H14B	0.369399	0.781038	0.745907	0.134*	
H14C	0.332975	0.746899	0.689537	0.134*	
C8	0.4238 (2)	0.6008 (10)	0.3455 (2)	0.0842 (17)	
H8A	0.442361	0.646829	0.309754	0.126*	
H8B	0.427952	0.449608	0.352592	0.126*	
H8C	0.392568	0.629283	0.334777	0.126*	
C10	0.4322 (2)	0.9641 (8)	0.3928 (3)	0.0794 (15)	
H10A	0.440539	1.046680	0.431129	0.119*	
H10B	0.451519	1.002048	0.357028	0.119*	
H10C	0.401251	0.993870	0.380523	0.119*	
N2	0.2841 (2)	−0.0247 (10)	0.4575 (3)	0.1095 (18)	
H2	0.267022	−0.043331	0.490969	0.131*	
C13	0.3886 (2)	1.0443 (9)	0.6502 (3)	0.0920 (19)	
H13A	0.408889	1.106044	0.618980	0.138*	
H13B	0.357876	1.072581	0.636557	0.138*	
H13C	0.394300	1.106719	0.692927	0.138*	
C20	0.2570 (2)	−0.4838 (11)	0.3587 (4)	0.0966 (19)	
H20	0.241637	−0.613249	0.362703	0.116*	
C17	0.3004 (2)	−0.1115 (11)	0.3471 (4)	0.0971 (19)	
H17	0.314885	0.020392	0.343305	0.117*	
C12	0.4451 (2)	0.7483 (11)	0.6794 (3)	0.0922 (18)	
H12A	0.450337	0.816345	0.721370	0.138*	
H12B	0.466707	0.799996	0.648434	0.138*	
H12C	0.448268	0.595631	0.684231	0.138*	
C19	0.2775 (2)	−0.4384 (12)	0.3005 (3)	0.0912 (18)	
H19	0.276520	−0.535037	0.265545	0.109*	
C18	0.2992 (2)	−0.2481 (13)	0.2956 (3)	0.098 (2)	
H18	0.313282	−0.211489	0.256542	0.117*	
H1	0.4585 (7)	0.975 (3)	0.5674 (9)	0.22 (5)*	

### Atomic displacement parameters (Å^2^)

**Table:**

	*U*^11^	*U*^22^	*U*^33^	*U*^12^	*U*^13^	*U*^23^
O1	0.101 (3)	0.082 (3)	0.078 (2)	−0.042 (2)	0.008 (2)	−0.013 (2)
C6	0.065 (3)	0.054 (3)	0.061 (3)	−0.006 (2)	0.005 (2)	0.002 (2)
C1	0.074 (3)	0.056 (3)	0.063 (3)	−0.012 (3)	0.006 (2)	−0.005 (2)
N3	0.086 (3)	0.110 (4)	0.091 (3)	0.011 (3)	0.016 (3)	0.009 (3)
C5	0.080 (3)	0.057 (3)	0.058 (3)	−0.008 (3)	0.001 (2)	0.002 (2)
C2	0.073 (3)	0.049 (3)	0.063 (3)	0.001 (2)	0.003 (2)	−0.002 (2)
C7	0.074 (3)	0.061 (3)	0.061 (3)	−0.009 (2)	0.011 (2)	0.001 (2)
C4	0.066 (3)	0.054 (3)	0.069 (3)	−0.006 (2)	0.004 (2)	0.003 (2)
C3	0.069 (3)	0.064 (3)	0.072 (3)	−0.008 (3)	0.009 (2)	0.007 (3)
C11	0.083 (3)	0.064 (3)	0.062 (3)	−0.002 (3)	0.006 (3)	−0.002 (2)
C16	0.094 (4)	0.074 (4)	0.087 (4)	0.003 (4)	−0.014 (4)	−0.012 (4)
C15	0.084 (4)	0.070 (3)	0.071 (3)	−0.014 (3)	−0.003 (3)	−0.014 (3)
N1	0.096 (4)	0.099 (4)	0.116 (4)	−0.026 (3)	0.007 (3)	0.020 (3)
C9	0.075 (3)	0.079 (4)	0.082 (3)	−0.002 (3)	0.013 (3)	−0.002 (3)
C14	0.109 (4)	0.096 (4)	0.064 (3)	0.011 (4)	0.025 (3)	0.004 (3)
C8	0.101 (4)	0.089 (4)	0.062 (3)	−0.016 (3)	0.000 (3)	−0.006 (3)
C10	0.089 (4)	0.066 (3)	0.083 (3)	−0.006 (3)	0.010 (3)	0.015 (3)
N2	0.115 (4)	0.109 (4)	0.105 (4)	−0.034 (4)	0.020 (3)	−0.002 (3)
C13	0.130 (5)	0.062 (4)	0.084 (4)	0.003 (3)	0.015 (4)	−0.011 (3)
C20	0.087 (4)	0.089 (5)	0.114 (5)	−0.017 (4)	0.009 (4)	0.006 (4)
C17	0.096 (5)	0.089 (5)	0.106 (5)	−0.009 (4)	−0.003 (4)	0.010 (4)
C12	0.094 (4)	0.111 (5)	0.071 (3)	0.007 (4)	−0.005 (3)	−0.006 (3)
C19	0.083 (4)	0.106 (5)	0.085 (4)	−0.014 (4)	0.013 (3)	−0.022 (4)
C18	0.087 (4)	0.127 (6)	0.081 (4)	−0.013 (4)	0.007 (3)	−0.002 (4)

### Geometric parameters (Å, º)

**Table:**

O1—C1	1.370 (6)	C9—H9A	0.9600
O1—H1	0.794 (15)	C9—H9B	0.9600
C6—C5	1.379 (6)	C9—H9C	0.9600
C6—C1	1.423 (6)	C14—H14A	0.9600
C6—C7	1.534 (7)	C14—H14B	0.9600
C1—C2	1.403 (7)	C14—H14C	0.9600
N3—C16	1.292 (8)	C8—H8A	0.9600
N3—C20	1.383 (8)	C8—H8B	0.9600
C5—C4	1.385 (7)	C8—H8C	0.9600
C5—H5	0.9300	C10—H10A	0.9600
C2—C3	1.377 (7)	C10—H10B	0.9600
C2—C11	1.561 (7)	C10—H10C	0.9600
C7—C8	1.530 (7)	N2—H2	0.8600
C7—C10	1.540 (7)	C13—H13A	0.9600
C7—C9	1.543 (7)	C13—H13B	0.9600
C4—C3	1.384 (7)	C13—H13C	0.9600
C4—C15	1.449 (7)	C20—C19	1.364 (9)
C3—H3	0.9300	C20—H20	0.9300
C11—C14	1.515 (7)	C17—C18	1.347 (9)
C11—C13	1.531 (7)	C17—H17	0.9300
C11—C12	1.555 (7)	C12—H12A	0.9600
C16—C17	1.369 (9)	C12—H12B	0.9600
C16—N2	1.386 (8)	C12—H12C	0.9600
C15—N1	1.253 (7)	C19—C18	1.351 (9)
C15—H15	0.9300	C19—H19	0.9300
N1—N2	1.396 (7)	C18—H18	0.9300
			
C1—O1—H1	136.7 (16)	C11—C14—H14A	109.5
C5—C6—C1	116.2 (4)	C11—C14—H14B	109.5
C5—C6—C7	122.0 (4)	H14A—C14—H14B	109.5
C1—C6—C7	121.7 (4)	C11—C14—H14C	109.5
O1—C1—C2	119.3 (4)	H14A—C14—H14C	109.5
O1—C1—C6	117.9 (4)	H14B—C14—H14C	109.5
C2—C1—C6	122.8 (4)	C7—C8—H8A	109.5
C16—N3—C20	117.4 (5)	C7—C8—H8B	109.5
C6—C5—C4	122.9 (4)	H8A—C8—H8B	109.5
C6—C5—H5	118.5	C7—C8—H8C	109.5
C4—C5—H5	118.5	H8A—C8—H8C	109.5
C3—C2—C1	116.7 (4)	H8B—C8—H8C	109.5
C3—C2—C11	121.5 (5)	C7—C10—H10A	109.5
C1—C2—C11	121.8 (4)	C7—C10—H10B	109.5
C8—C7—C6	111.8 (4)	H10A—C10—H10B	109.5
C8—C7—C10	107.0 (4)	C7—C10—H10C	109.5
C6—C7—C10	111.6 (4)	H10A—C10—H10C	109.5
C8—C7—C9	106.1 (4)	H10B—C10—H10C	109.5
C6—C7—C9	110.0 (4)	C16—N2—N1	122.6 (6)
C10—C7—C9	110.2 (4)	C16—N2—H2	118.7
C3—C4—C5	118.3 (4)	N1—N2—H2	118.7
C3—C4—C15	119.6 (5)	C11—C13—H13A	109.5
C5—C4—C15	122.0 (5)	C11—C13—H13B	109.5
C2—C3—C4	123.1 (5)	H13A—C13—H13B	109.5
C2—C3—H3	118.5	C11—C13—H13C	109.5
C4—C3—H3	118.5	H13A—C13—H13C	109.5
C14—C11—C13	106.7 (5)	H13B—C13—H13C	109.5
C14—C11—C12	107.6 (4)	C19—C20—N3	122.5 (6)
C13—C11—C12	111.2 (5)	C19—C20—H20	118.7
C14—C11—C2	111.5 (4)	N3—C20—H20	118.7
C13—C11—C2	110.3 (4)	C18—C17—C16	120.1 (7)
C12—C11—C2	109.5 (4)	C18—C17—H17	120.0
N3—C16—C17	122.2 (6)	C16—C17—H17	120.0
N3—C16—N2	119.8 (7)	C11—C12—H12A	109.5
C17—C16—N2	118.0 (6)	C11—C12—H12B	109.5
N1—C15—C4	121.9 (6)	H12A—C12—H12B	109.5
N1—C15—H15	119.1	C11—C12—H12C	109.5
C4—C15—H15	119.1	H12A—C12—H12C	109.5
C15—N1—N2	118.8 (6)	H12B—C12—H12C	109.5
C7—C9—H9A	109.5	C18—C19—C20	117.6 (6)
C7—C9—H9B	109.5	C18—C19—H19	121.2
H9A—C9—H9B	109.5	C20—C19—H19	121.2
C7—C9—H9C	109.5	C17—C18—C19	120.1 (6)
H9A—C9—H9C	109.5	C17—C18—H18	119.9
H9B—C9—H9C	109.5	C19—C18—H18	119.9
			
C5—C6—C1—O1	177.4 (4)	C15—C4—C3—C2	175.1 (5)
C7—C6—C1—O1	−2.6 (7)	C3—C2—C11—C14	−3.1 (7)
C5—C6—C1—C2	−1.4 (7)	C1—C2—C11—C14	179.9 (5)
C7—C6—C1—C2	178.6 (5)	C3—C2—C11—C13	−121.5 (5)
C1—C6—C5—C4	0.4 (7)	C1—C2—C11—C13	61.6 (6)
C7—C6—C5—C4	−179.6 (5)	C3—C2—C11—C12	115.8 (6)
O1—C1—C2—C3	−177.8 (4)	C1—C2—C11—C12	−61.1 (6)
C6—C1—C2—C3	1.0 (7)	C20—N3—C16—C17	2.1 (9)
O1—C1—C2—C11	−0.7 (7)	C20—N3—C16—N2	−177.6 (6)
C6—C1—C2—C11	178.1 (4)	C3—C4—C15—N1	−172.4 (6)
C5—C6—C7—C8	1.9 (7)	C5—C4—C15—N1	4.0 (8)
C1—C6—C7—C8	−178.1 (5)	C4—C15—N1—N2	177.9 (5)
C5—C6—C7—C10	121.7 (5)	N3—C16—N2—N1	166.7 (6)
C1—C6—C7—C10	−58.3 (6)	C17—C16—N2—N1	−13.1 (9)
C5—C6—C7—C9	−115.6 (5)	C15—N1—N2—C16	−175.4 (6)
C1—C6—C7—C9	64.4 (6)	C16—N3—C20—C19	−0.5 (9)
C6—C5—C4—C3	1.0 (7)	N3—C16—C17—C18	−2.5 (10)
C6—C5—C4—C15	−175.5 (5)	N2—C16—C17—C18	177.3 (6)
C1—C2—C3—C4	0.5 (7)	N3—C20—C19—C18	−0.8 (10)
C11—C2—C3—C4	−176.6 (5)	C16—C17—C18—C19	1.1 (10)
C5—C4—C3—C2	−1.5 (8)	C20—C19—C18—C17	0.5 (10)

### Hydrogen-bond geometry (Å, º)

**Table:**

*D*—H···*A*	*D*—H	H···*A*	*D*···*A*	*D*—H···*A*
N2—H2···N3^i^	0.86	2.23	3.062 (8)	162

Symmetry code: (i) −*x*+1/2, −*y*−1/2, −*z*+1.
